# From Suspected Congenital Cytomegalovirus Infection to Malan Syndrome: Delayed Genetic Diagnosis Due to Diagnostic Anchoring

**DOI:** 10.3390/diseases14060191

**Published:** 2026-05-28

**Authors:** Gordana Kovacevic, Sanja Cirkovic, Gordana Petrovic, Maja Stanojevic, Tanja Lalic, Nikola Ilic, Slavica Ostojic, Marina Siljic, Biljana Alimpic, Milanka Tesic, Predrag Ilic, Jovana Krstic, Jana Cirkovic, Adrijan Sarajlija

**Affiliations:** 1 Department of Neurology, Mother and Child Health Care Institute of Serbia “Dr Vukan Čupić”, 11070 Belgrade, Serbia; slavica.ostojic@imd.org.rs; 2 Faculty of Medicine, University of Belgrade, 11070 Belgrade, Serbia; maja.stanojevic@med.bg.ac.rs (M.S.); marina.siljic@med.bg.ac.rs (M.S.); predrag.ilic70@yahoo.com (P.I.); adrijan.sarajlija@imd.org.rs (A.S.); 3 Laboratory of Medical Genetics, Mother and Child Health Care Institute of Serbia “Dr Vukan Čupić”, 11070 Belgrade, Serbia; sanja.s.cirkovic@gmail.com (S.C.); tanja.lalic68@gmail.com (T.L.); 4 Department of Immunology, Mother and Child Health Care Institute of Serbia “Dr Vukan Čupić”, 11070 Belgrade, Serbia; gordana.petrovic.im@gmail.com; 5 Institute of Microbiology and Immunology, Dr Subotica 1, 11000 Belgrade, Serbia; 6 Division of Clinical Genetics, Mother and Child Health Care Institute of Serbia “Dr Vukan Čupić”, 11070 Belgrade, Serbia; ilicnikola91@gmail.com; 7 Day Hospital Care, Mother and Child Health Care Institute of Serbia “Dr Vukan Čupić”, 11070 Belgrade, Serbia; biljana.alimpic@imd.org.rs; 8 Bone Marrow Transplant Department, Mother and Child Health Care Institute of Serbia “Dr Vukan Čupić”, 11070 Belgrade, Serbia; milanka.tesic@imd.org.rs; 9 Department of Urology, Mother and Child Health Care Institute of Serbia “Dr Vukan Čupić”, 11070 Belgrade, Serbia; 10 Pediatric Clinic, Mother and Child Health Care Institute of Serbia “Dr Vukan Čupić”, 11070 Belgrade, Serbia; jovana.krstic@imd.org.rs (J.K.); jana.cirkovic.10@gmail.com (J.C.)

**Keywords:** Malan syndrome, chromosomal microarray analysis, 19p13.13 microdeletion, congenital cytomegalovirus, diagnostic anchoring, developmental delay

## Abstract

**Background:** Diagnostic anchoring to a presumed infectious etiology may delay recognition of underlying genetic disorders in children with neurodevelopmental impairment. **Case presentation:** A case of a child with sensorineural hearing loss, visual impairment, and developmental delay is reported; cytomegalovirus (CMV) infection was identified at 6 months of age based on positive serology and detection of viral DNA in serum and urine. Given the timing of testing, congenital CMV infection (cCMV) could not be definitively confirmed. Antiviral therapy with valganciclovir was administered. Despite antiviral treatment, severe neurodevelopmental impairment and hearing loss persisted, associated with facial dysmorphism, bilateral cryptorchidism, pectus excavatum, and optic nerve hypoplasia, findings not fully attributable to CMV infection. Brain magnetic resonance imaging (MRI) showed nonspecific findings. Chromosomal microarray analysis (CMA) performed at 4.5 years of age identified a heterozygous 908 kb de novo microdeletion at 19p13.2p13.13 containing *NFIX* (MIM *164005) and other morbid genes. The de novo variant was confirmed by parental testing, and the unifying genetic diagnosis of *NFIX*-related Malan syndrome (MIM#614753) was established. **Conclusions:** This case emphasizes the importance of reconsidering the initial diagnosis when the clinical phenotype is not fully consistent with an infectious etiology. Early genomic testing, including CMA, may facilitate timely recognition of underlying genetic syndromes in children with complex neurodevelopmental presentations.

## 1. Introduction

Chromosomal microarray analysis (CMA) is commonly used as a first-tier genetic test in children with developmental delay, intellectual disability, and congenital anomalies. It has significantly improved the diagnostic yield in patients with neurodevelopmental disorders of unclear cause [1].

However, in clinical practice, early findings that appear to explain the clinical presentation may lead to narrowing of the diagnostic perspective. Infections such as cytomegalovirus (CMV) infection can present with features overlapping those of genetic disorders, including hearing loss and developmental delay [2]. In certain cases, once an infectious cause is identified, further investigation may be limited, even when the clinical phenotype is complex and cannot be fully explained by infection. This may lead to delayed recognition of the underlying genetic disorder.

This report describes a male child initially evaluated for suspected congenital CMV (cCMV) infection. His severe and complex clinical manifestations prompted delayed genetic evaluation, ultimately leading to the detection of a causative 19p13.2p13.13 microdeletion. This case highlights how an early presumed infectious diagnosis may influence clinical judgment and delay genetic testing, even when the phenotype suggests an alternative underlying condition.

## 2. Case Presentation

The patient is the parents’ second child born from an uneventful, full-term pregnancy and delivery. Birth parameters were within normal limits: weight 3000 g, length 50 cm, and head circumference 35 cm. The Apgar score was 9 at the first minute. There was no reported family history of genetic or neurological disorders.

At one month of age, the parents noted reduced responsiveness to auditory stimuli and increased muscle tone, prompting initiation of physical therapy. At six months, the child was admitted for further evaluation. On examination, his body weight (BW) was 7.5 kg (between 0 and +1 SD), length 73 cm (+2 SD), and head circumference 43.5 cm (0 SD). The patient demonstrated poor responsiveness to external stimuli, poor visual contact, and absence of the auropalpebral reflex. Hypertonia of the extremities was present. The child was unable to attain a sitting position on traction, but he was able to lift and turn his head while he was in prone position. Deep tendon reflexes were symmetric.

Additional clinical findings included divergent strabismus, bilateral cryptorchidism, and rectal stenosis. The results of laboratory investigations, including liver function tests, lactate levels, and complete blood count, were within normal limits. Abdominal ultrasound was unremarkable, and fundoscopic examination revealed no abnormalities. Flash visual evoked potentials (fVEPs) demonstrated prolonged latencies, consistent with delayed visual pathway conduction.

Audiological assessment revealed bilateral sensorineural hearing loss. Brain magnetic resonance imaging (MRI) showed bilateral optic nerve hypoplasia along with nonspecific abnormalities, including a subarachnoid cyst and mild ventricular dilatation.

Given the presence of hearing impairment and psychomotor developmental delay, serological testing for intrauterine infections was performed. The results demonstrated positive IgM and IgG antibodies to CMV, and polymerase chain reaction (PCR) analysis confirmed the presence of CMV DNA in both blood and urine samples. Despite it being beyond the neonatal period, antiviral therapy with valganciclovir at a dose of 16 mg/kg BW twice daily was initiated due to significant hearing loss and developmental delay. The treatment was administered over six months and was completed without complications.

However, severe global developmental delay and hearing impairment persisted without improvement following antiviral therapy. Independent sitting was achieved at four years of age, and assisted ambulation with a few steps at five years. The child remained nonverbal and showed limited interaction with the environment. Severe sensorineural hearing loss was initially managed with hearing amplification, but due to insufficient benefit, cochlear implantation was subsequently performed.

Poor weight gain was observed from early infancy, necessitating a high-calorie nutritional regimen. Feeding difficulties were present, including impaired chewing and swallowing of solid foods. Chronic constipation was also noted. Rectal stenosis necessitated regular rectal catheterization during the first 15 months of life. Bilateral orchidopexy was performed at 20 months of age.

At five years of age, his body weight was 16.3 kg (between −1 and −2 SD), his height was 125.3 cm (+2 SD), and his body mass index was 10.4 (−3 SD). His head circumference was 51 cm (0–1 SD).

**Figure 1:**
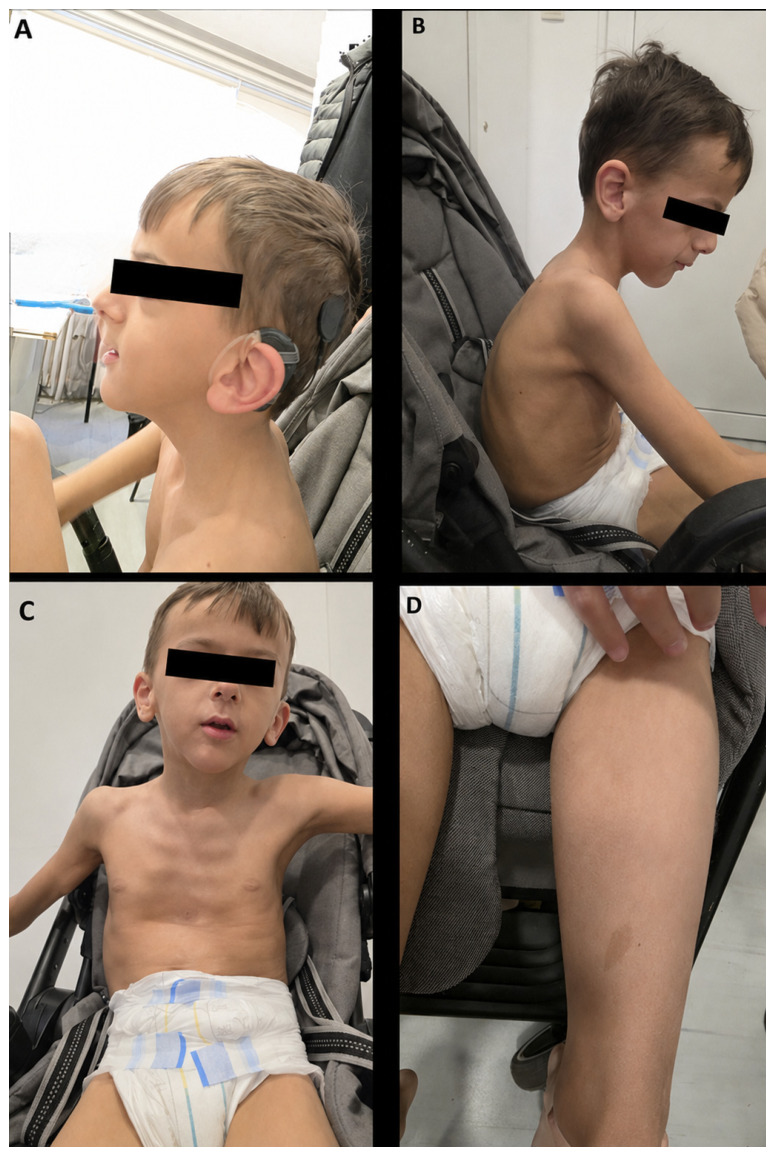
Phenotypic features of the patient with Malan syndrome. (**A**) Facial profile with elongated facial morphology and a cochlear implant in situ. (**B**,**C**) Asthenic body habitus with marked leanness and reduced subcutaneous tissue. (**D**) Focal skin hyperpigmentation on the thigh, consistent with the variable phenotypic spectrum.

Follow-up examinations revealed additional dysmorphic features, including an elongated facial appearance and large ears, chest wall deformity consistent with pectus excavatum, thoracic kyphosis, and generalized muscle hypotrophy (Figure 1). 

**Figure 2:**
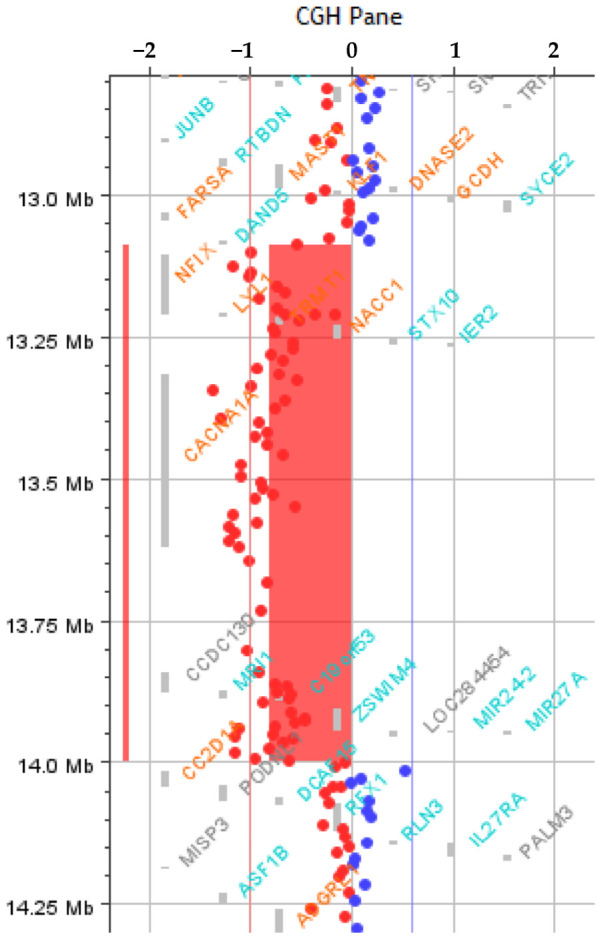
High-resolution chromosomal microarray plot demonstrating the pathogenic 908 kb deletion at 19p13.2p13.13 encompassing key genes. Red dots represent the patient’s log_2_ ratio values (indicating copy number loss), while blue dots represent the reference DNA. The red bar highlights the 908 kb deletion.

Given the presence of clinical features that go beyond the typical phenotype of cCMV infection, CMA was performed on DNA extracted from the patient’s blood at the age of 4 years and 6 months, using the Agilent SurePrint G3 Human CGH microarray kit (4 × 180,000 probes, 60 bp each) according to the manufacturer’s protocol (Agilent Technologies, Santa Clara, CA, USA). High-resolution CMA revealed an aberrant male profile with a heterozygous interstitial 908 kb deletion at chromosome 19: arr[GRCh37] 19p13.2p13.13 (13,089,757_13,997,370) × 1 [3]. The deletion encompasses 13 protein-coding genes, five of which are OMIM morbid genes: *NFIX* (MIM *164005), *TRMT1* (MIM *611669), *NACC1* (MIM *610672), *CACNA1A* (MIM *601011), and *LYL1* (MIM *151440) (Figure 2). The variant was classified as pathogenic according to ACMG/ClinGen technical standards for constitutional copy number loss (initial criteria: 1A, 2A, 3A, 5G) [4]. Subsequent CMA testing of both parents confirmed that the deletion occurred de novo (final criteria: 1A, 2A, 3A, 5A) [4]. This finding is diagnostic of *NFIX*-related Malan syndrome (MIM #614753) caused by 19p13.13 microdeletion (MIM #613638).

As part of the retrospective evaluation, dried blood spots (DBSs) from the patient’s archived Guthrie card were tested for the presence of CMV DNA. The Guthrie card was obtained from a centralized repository where they are stored dry, in the dark, at room temperature. DNA extraction was performed using the QIAamp DNA Mini Kit (Qiagen, Hilden, Germany), according to the manufacturer’s instructions, whereas CMV detection was done using the GeneProof Cytomegalovirus (CMV) PCR Kit (GeneProof, Brno, Czech Republic). The obtained result was negative.

## 3. Discussion

Congenital cytomegalovirus infection is the most common infectious cause of sensorineural hearing loss and neurodevelopmental impairment in early childhood. It is frequently unrecognized at birth, as merely 10–15% of affected newborns are symptomatic [5,6]. The prevalence of cCMV is estimated at 0.6–0.7% (~1 in 200 live births) in developed countries [7,8,9]. A comprehensive meta-analysis conducted in 2021 revealed a global prevalence rate of 0.67%. This rate was approximately three times higher in low- and middle-income countries, at 1.42%, compared to 0.48% in high-income countries [10].

The range of clinical manifestations in cCMV infection is quite broad, spanning from asymptomatic cases to those with significant neurological and multiple organ system involvement. Common manifestations include sensorineural hearing loss, developmental delay, microcephaly, intracranial calcifications, and chorioretinitis [6].

The risk of neurological sequelae is high, especially among symptomatic infants, of whom more than 50% develop permanent neurological impairment. Common neurological sequelae include developmental delay, intellectual impairment, visual impairment, and seizures [11].

Hearing loss is the most common complication, affecting both symptomatic newborns (about 35%) and a smaller proportion (7–10%) of newborns who were asymptomatic at birth [8].

Antiviral therapy (ganciclovir or valganciclovir) has been shown to improve hearing and neurodevelopmental outcomes in selected symptomatic infants with cCMV infection, particularly when initiated early [5,12].

When CMV infection is diagnosed beyond the neonatal period, differentiation between congenital and postnatal infection may be challenging [12]. This can complicate diagnostic evaluation, especially when additional genetic syndromes are present.

A positive CMV PCR in serum and urine at 6 months of age confirmed CMV infection in the present case; however, due to the delayed timing of testing it was not possible to distinguish between congenital and postnatal acquisition. Therefore, cCMV infection could not be definitively established in this patient. According to current diagnostic criteria, confirmation of cCMV requires virological testing within the first 2–3 weeks of life [5,12].

Furthermore, by retrospective testing using PCR on the patient’s archived Guthrie card, cCMV infection could not be confirmed, since the result was negative. However, this finding should be interpreted cautiously, as a negative DBS PCR result does not definitively exclude cCMV infection. In spite of some possible limitations of this method, considering viral DNA stability under storage conditions and the potential risk of cross-contamination, testing neonatal DBS samples continues to be a valid and useful approach for both screening and clinical purposes [13,14,15,16,17].

Although CMV infection was confirmed, several clinical features in the patient were not fully explained by either congenital or acquired CMV. These included multiple congenital anomalies (bilateral cryptorchidism, rectal stenosis), dysmorphic facial features, skeletal abnormalities (pectus excavatum, kyphosis), and disproportionate growth. Collectively, this phenotype could itself have justified earlier genetic evaluation. In addition, neuroimaging findings, such as a subarachnoid cyst and mild ventricular dilatation, were nonspecific and insufficient to account for the global neurodevelopmental impairment.

The persistence of severe developmental delay and hearing impairment despite antiviral treatment, along with atypical clinical features, raised suspicion of an additional underlying etiology and prompted further genetic evaluation.

Current guidelines recommend CMA as a first-tier diagnostic tool in patients with developmental delay, intellectual disability, and multiple congenital anomalies. Its high resolution enables the detection of submicroscopic copy number variants that are not identifiable by conventional cytogenetic methods [1].

The present case illustrates the diagnostic utility of CMA even when an alternative etiology, such as a presumed congenital infection, initially appears sufficient. The identified 19p13.2p13.13 microdeletion established the unifying diagnosis of *NFIX*-related Malan syndrome (MIM #614753) within the broader spectrum of 19p13.13 microdeletion syndrome (MIM #613638). This rare autosomal dominant condition is typically caused by a de novo deletion encompassing multiple genes, including *NFIX* (MIM *164005) and *CACNA1A* (MIM *601011) [18]. *NFIX* encodes a transcription factor involved in the regulation of genes essential for brain development, glial differentiation, skeletal development, and postnatal growth [19]. Haploinsufficiency of *NFIX* is considered the key molecular mechanism underlying Malan syndrome; thus, *NFIX*-containing 19p13.13 microdeletions are generally regarded as part of the Malan syndrome spectrum, although the phenotype may be modified by the involvement of additional genes. Approximately one quarter of individuals with Malan syndrome harbor a heterozygous microdeletion of 19p13.13 encompassing the *NFIX* gene, highlighting copy number variation as a significant pathogenic mechanism in this disorder [18]. Importantly, the relatively small size of the detected deletion (~900 kb) underscores the diagnostic sensitivity of CMA, as such submicroscopic imbalances would be missed by conventional karyotyping. This finding further highlights the value of high-resolution genomic testing in patients with complex phenotypes, even when an alternative etiology appears plausible.

Clinically, Malan syndrome is most consistently characterized by facial dysmorphism, in addition to developmental delay and intellectual disability. Typical features include a long or triangular face, prominent forehead, depressed nasal bridge, deep-set eyes with down-slanting palpebral fissures, short nose, anteverted nares, long philtrum, small mouth with thin upper vermillion, and prominent chin [20]. The patient exhibited several of these characteristic facial features, along with skeletal abnormalities such as thoracolumbar scoliosis and pectus deformity, as well as visual involvement—findings that are commonly reported in individuals with Malan syndrome.

Although Malan syndrome is traditionally classified as an overgrowth disorder, growth parameters are variable. Birth length is typically within the normal range, and postnatal overgrowth is observed in only a subset of patients with 19p13.13 deletions involving *NFIX* [20,21]. Macrocephaly appears to be a more consistent feature. In the patient, the absence of marked overgrowth did not exclude the diagnosis but contributed to diagnostic uncertainty and delay.

Nevertheless, the coexistence of developmental delay, dysmorphic facial features, multiple congenital anomalies, and hearing impairment suggested a complex neurodevelopmental phenotype, warranting earlier evaluation using a high-throughput genomic approach such as CMA.

Taken together, the genetic finding and the partial but significant phenotypic overlap support interpreting this case as *NFIX*-related Malan syndrome within a broader 19p13.13 contiguous microdeletion syndrome. The overall phenotype likely reflects the combined effects of multiple deleted genes.

The adjacent loss of *CACNA1A*, which encodes the α1A subunit of P/Q-type voltage-gated calcium channels critical for synaptic transmission and neuronal excitability, may contribute to more severe neurodevelopmental impairment and increased seizure susceptibility [18,22,23]. However, the patient has not exhibited clinical seizures to date, and electroencephalographic evaluation has not demonstrated clear epileptiform discharges. Additional genes within the deleted region likely contribute to the phenotype. Haploinsufficiency of *NACC1* (MIM *610672) has been associated with severe developmental delay, epilepsy, and feeding difficulties, while *TRMT1* (MIM *611669) may further exacerbate intellectual disability [24]. These genes are located within the smallest region of overlap identified in earlier reports, supporting a contiguous gene deletion mechanism. Other genes in the interval (*LYL1*, *STX10*, *IER2*, *MRI1*, *C19orf53*, *ZSWIM4*, *NANOS3*, and *BRME1*) are not clearly associated with dominant phenotypes; however, a cumulative haploinsufficiency effect likely contributes to the overall clinical severity [25].

Hearing impairment in Malan syndrome is considered uncommon but has been previously reported, including cases of bilateral moderate-to-severe sensorineural hearing loss [20]. In adults with Malan syndrome, hearing impairment, including sensorineural, conductive, and progressive forms, has been described in approximately 20% of patients, while altered auditory processing and hypersensitivity to noise appear even more frequently (46.4%) [26].

The persistence of sensorineural hearing loss despite antiviral treatment suggested that hearing impairment may represent an underrecognized feature within the phenotypic spectrum of Malan syndrome. However, as the CMV infection could not be definitively classified as congenital or postnatal, it remains unclear to what extent the hearing loss was related to the CMV infection, Malan syndrome, or both.

Therefore, the diagnosis of Malan syndrome, established by CMA through detection of a pathogenic alteration involving *NFIX*, provides a unifying explanation for the patient’s phenotype. Several clinical features—including profound developmental delay, sensorineural hearing loss, dysmorphic features, tall stature, and multiple congenital anomalies—extend beyond the expected clinical spectrum of cCMV infection.

The clinical characteristics of cCMV infection, *NFIX*-related Malan syndrome and the present patient are summarized in Table 1.

This case illustrates the complexity of etiological assessment in infants presenting with neurodevelopmental impairment and multisystem involvement, particularly when an infectious agent such as CMV is identified. Although CMV may have contributed to some findings, the disproportionate severity and multisystem involvement prompted further genetic evaluation, revealing an underlying chromosomal disorder.

The identification of a 19p13.2p13.13 microdeletion provided an explanation for the observed phenotype and supports the interpretation that *NFIX*-related Malan syndrome represents the primary etiology. In contrast, CMV infection in this case most likely represents either a postnatal infection or an unconfirmed congenital infection with limited contribution to the overall clinical phenotype. Rather than representing a straightforward dual pathology, this report demonstrates how the presence of a plausible infectious finding can lead to diagnostic anchoring and delay consideration of an underlying genetic diagnosis. It also exemplifies how genomic and infectious etiologies may coexist or be sequentially identified, but careful phenotypic reassessment remains essential to avoid premature diagnostic closure. Finally, this report reinforces the role of CMA as an essential early diagnostic tool and highlights the need for a multidisciplinary approach in evaluating children with complex neurodevelopmental presentations.

**Figure 3:**
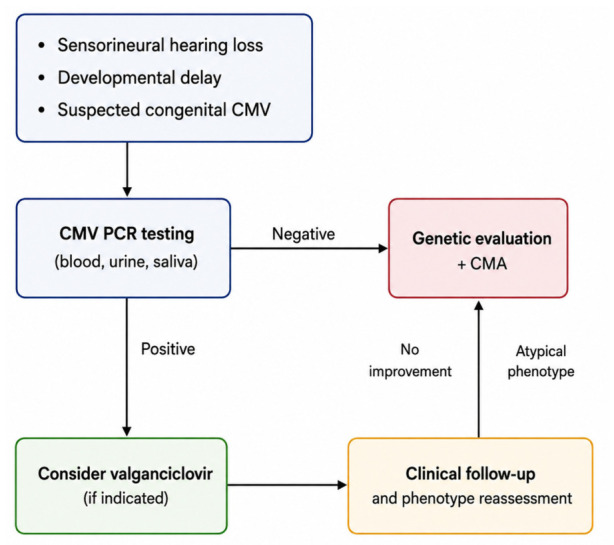
Proposed diagnostic workflow for children with sensorineural hearing loss, developmental delay, and suspected CMV infection. Abbreviations: CMV, cytomegalovirus; CMA, chromosomal microarray analysis; PCR, polymerase chain reaction.

The findings from this case support careful phenotypic reassessment throughout follow-up in children presenting with hearing impairment, developmental delay, and suspected CMV infection. While evaluation for cCMV remains essential, the presence of congenital anomalies, dysmorphic features, disproportionate growth, severe developmental impairment, atypical neuroimaging findings, or clinical evolution not fully explained by CMV should raise suspicion of an underlying genetic disorder. In such cases, early parallel genetic investigation, particularly CMA, should be considered. A multidisciplinary and phenotype-driven diagnostic approach may help avoid diagnostic anchoring and improve diagnostic accuracy in complex neurodevelopmental disorders (Figure 3).

An earlier diagnosis of *NFIX*-related Malan syndrome established by CMA could have improved several aspects of long-term management and genetic counseling. Valganciclovir therapy for presumed cCMV infection would likely still have been justified because of the presence of the significant sensorineural hearing loss, a common manifestation of cCMV infection. However, earlier recognition of the underlying genetic syndrome could have led to earlier multidisciplinary developmental follow-up, individualized rehabilitation planning, and reduced diagnostic uncertainty for both clinicians and the family. Confirmation of the deletion would have enabled accurate genetic counseling. Although the recurrence risk is considered low because of the apparently de novo origin of the CNV, it is not negligible due to rare reported cases of parental gonadal mosaicism involving *NFIX* alterations [27,28]. Therefore, prenatal CMA testing may be offered in future pregnancies following appropriate genetic counseling. Overall, this case highlights how diagnostic anchoring to an infectious etiology may delay recognition of an underlying genetic syndrome, and it supports the role of early genomic testing in complex neurodevelopmental phenotypes.

## 4. Conclusions

This case indicates the need for high-resolution genomic testing for complex neurodevelopmental disorders, even when an alternative diagnosis is apparent at first glance. Detection of CMV infection in infancy should not preclude genetic evaluation in children with severe or atypical neurodevelopmental phenotypes. Timely CMA is essential to avoid delayed diagnosis of underlying genetic syndromes and to ensure comprehensive patient care and accurate genetic counseling.

## Figures and Tables

**Table 1:** Comparison of clinical features in congenital CMV infection, *NFIX*-related Malan syndrome, and the present patient.

Clinical Feature	Congenital CMV Infection	*NFIX*-Related Malan Syndrome	Present Patient
Sensorineural hearing loss	Common	Occasionally reported	Present
Developmental delay	Common moderate to severe	Common—moderate	Severe
Microcephaly/macrocephaly	Common, microcephaly	Variable—macrocephaly	Normal head circumference
Intracranial abnormalities	Common calcifications, ventriculomegaly, migrational abnormalities	Variable—corpus callosum and cerebellar vermis hypoplasia	Ventricular dilatation, arachnoid cyst
Visual impairment	Possible (chorioretinitis)	Common—strabismus, optic hypoplasia	Strabismus, optic hypoplasia
Dysmorphic features	Uncommon	Common—frontal bossing, low-set ears, elongated face	Elongated face, large ears
Congenital anomalies	Rare	Common	Present
Cryptorchidism	Rare	Reported	Present
Gastrointestinal anomalies	Rare	Occasional—vomiting, constipation	Rectal stenosis, constipation
Growth abnormalities	Intrauterine growth retardation common	Overgrowth	Tall stature
Slender habitus	Absent	Present	Present
Skeletal abnormalities	Rare	Reported—pectus excavatum	Pectus excavatum, kyphosis
Feeding difficulties	Possible	Swallowing, bulbar problems	Difficulties swallowing solid food

## Data Availability

The data presented in this study are not publicly available due to ethical and privacy restrictions related to sensitive patient clinical and genetic data obtained from hospital medical records.
